# Baseline synovitis–tenosynovitis is associated with remission in early rheumatoid arthritis, but discordance with disease activity is a changeable state

**DOI:** 10.1093/rheumatology/keaf098

**Published:** 2025-02-17

**Authors:** Rudresh R Shukla, Richard J Wakefield, Pauline Ho, Ai Lyn Tan, Paul Emery, Darren Plant, Maya H Buch

**Affiliations:** Centre for Musculoskeletal Research, University of Manchester, Manchester, UK; NIHR Manchester Biomedical Research Centre, Manchester University Hospitals NHS Foundation Trust, Manchester, UK; Leeds Institute of Rheumatic and Musculoskeletal Medicine, School of Medicine, University of Leeds, Leeds, UK; NIHR Leeds Biomedical Research Centre, Leeds Teaching Hospitals NHS Trust, Leeds, UK; Centre for Musculoskeletal Research, University of Manchester, Manchester, UK; NIHR Manchester Biomedical Research Centre, Manchester University Hospitals NHS Foundation Trust, Manchester, UK; Leeds Institute of Rheumatic and Musculoskeletal Medicine, School of Medicine, University of Leeds, Leeds, UK; NIHR Leeds Biomedical Research Centre, Leeds Teaching Hospitals NHS Trust, Leeds, UK; Leeds Institute of Rheumatic and Musculoskeletal Medicine, School of Medicine, University of Leeds, Leeds, UK; NIHR Leeds Biomedical Research Centre, Leeds Teaching Hospitals NHS Trust, Leeds, UK; Centre for Musculoskeletal Research, University of Manchester, Manchester, UK; NIHR Manchester Biomedical Research Centre, Manchester University Hospitals NHS Foundation Trust, Manchester, UK; Centre for Musculoskeletal Research, University of Manchester, Manchester, UK; NIHR Manchester Biomedical Research Centre, Manchester University Hospitals NHS Foundation Trust, Manchester, UK; Leeds Institute of Rheumatic and Musculoskeletal Medicine, School of Medicine, University of Leeds, Leeds, UK

**Keywords:** rheumatoid, ultrasound, discordance, transitions, biologic

## Abstract

**Objectives:**

The objectives of this study were to investigate the association between baseline joint-complex inflammation [power Doppler–detected joint synovitis (PDUS) and/or tenosynovitis (PDTS)] and remission in treatment-naïve, new-onset RA patients and to evaluate concordance and discordance states between clinical disease activity and power Doppler US and transition between these states longitudinally.

**Methods:**

At baseline, treatment-naïve early RA patients from a randomized controlled trial were categorized according to dominant hand PDUS and/or PDTS presence into four groups (PDUS+PDTS+, PDUS+PDTS−, PDUS−PDTS+, PDUS−PDTS−). Longitudinally, patients were grouped based on both clinical DAS and PDUS presence into: DAS+PDUS+ (DAS28-ESR > 2.6, PDUS > 0), DAS+PDUS− (DAS28-ESR > 2.6, PDUS = 0), DAS−PDUS+ (DAS28ESR ≤ 2.6, PDUS > 0) and DAS−PDUS− (DAS28ESR ≤ 2.6, PDUS = 0). Bayesian logistic regression analysis was applied.

**Results:**

Baseline PDUS+PDTS+ was associated with week 24 remission (posterior estimate = 1.41, credible interval = 0.16–2.65). At baseline diagnosis, 68% were DAS+PDUS+ and 32% DAS+PDUS−. Early transition from DAS+PDUS+ to DAS+PDUS− (32% at week 12) occurred. Overall proportions with DAS+PDUS− remained unchanged (43% at week 24); however, individual membership of this group changed over time, with only 41% at baseline remaining DAS+PDUS− through to week 48.

**Conclusion:**

In new-onset RA, baseline joint-complex power Doppler US associates with week 24 remission. DAS+PDUS− emerges early but, like DAS+PDUS+ and DAS−PDUS−, is a dynamic state, indicating opportunity for therapeutic targeting. Understanding the basis for these states can aid stratification and personalized treatment strategies.

## Introduction

RA is a heterogeneous disease, and individuals with RA may respond differently to therapies, leading to variable outcomes and different trajectories of disease [1]. Clinical measures used to assess response are limited by their lack of agreement [2–4] and poor correlation with individual components [5, 6].

Several studies have highlighted the prognostic and predictive role of musculoskeletal ultrasound (MSUS)-detected tenosynovitis and joint synovitis across the RA continuum [7–12]. MSUS findings, however, may be discrepant with the clinical evaluation—it can detect power Doppler US synovitis (PDUS) indicative of active inflammation in the absence of clinically swollen joints and vice versa [7, 13–15]. Persistence of measured disease activity in the absence of objective measures of inflammation has been attributed to chronic pain states and is considered largely unmodifiable by DMARDs [16]. Whether and how such discrepancy is maintained longitudinally and in a treatment-naïve cohort is not known.

In the current study, we undertook post-hoc analysis of a randomized controlled trial of treatment-naïve, early RA, to (i) investigate the association between baseline joint-complex inflammation [PDUS-detected joint synovitis (PDUS) and/or tenosynovitis (PDTS)] and subsequent remission, and (ii) to evaluate the concordance/discordance states between clinical disease activity and power Doppler US and the change between these states over time.

## Methods

### Patients and study design

‘VEDERA’ (Very Early *vs* Delayed Etanercept in patients with early RA) was a single-centre, phase IV, open-label, two-arm trial that randomized 120 participants with treatment-naïve, new-onset early RA and DAS28-ESR ≥ 3.2 to either first-line etanercept + MTX (ETN + MTX) or a MTX treat-to-target (MTX-TT) regimen with escalation to ETN + MTX if not in DAS28ESR remission at week 24. As per the VEDERA trial eligibility, if participants were seronegative (RF and ACPA negative), the presence of PDUS (at least grade 1 or more) was required. The primary trial results have been published [17].

### Imaging assessment

Clinical and MSUS assessments were performed at baseline, weeks 12, 24 and 48. MSUS assessments were completed on dominant hand (MCP joints 1–5, wrist radiocarpal/intercarpal joints, flexor tendons 1–5 and extensor carpi ulnaris tendon) and/or clinically symptomatic joints. Joint synovitis and tenosynovitis were assessed using a semiquantitative (0–3) score of grey-scale (GS) and power Doppler US. MSUS assessments were performed by an experienced ultrasonographer blinded to treatment allocation.

Patients were stratified at baseline by the presence/absence of PDUS (ie joint synovitis) and/or PDTS (tenosynovitis) into four groups—power Doppler US present at both levels (PDUS+PDTS+), PDUS only (PDUS+PDTS−), PDTS only (PDUS−PDTS+), power Doppler US absent at both levels (PDUS−PDTS−).

### Defining concordance and discordance disease activity states

DAS28ESR was used to assess clinical disease activity state in keeping with the original trial primary end point. Clinically active disease was defined as DAS28-ESR > 2.6 and clinical remission as DAS28-ESR ≤ 2.6. Presence of power Doppler US joint/tendon (PDUS/PDTS, respectively) was defined as total power Doppler US score > 0. Patients were grouped into the following categories based on clinical disease activity and presence/absence of power Doppler US (with PDUS and PDTS grouped and analysed separately).

- DAS+PD+ = clinically active and power Doppler US present
- DAS+PD− = clinically active and power Doppler US absent
- DAS−PD+ = clinical remission and power Doppler US present
- DAS−PD− = clinical remission and power Doppler US absent.

### Statistical analysis

At baseline, clinical and GS features were compared within the four PDUS– and/or PDTS–defined groups using the Kruskal–Wallis rank sum test and Pearson’s chi-squared test with a significance threshold of 5% (*P* < 0.05).

To examine the association between baseline PDUS and/or PDTS and subsequent clinical remission at weeks 12, 24 and 48, a Bayesian logistic regression model using weakly informative priors was defined using the Rstanarm package [18]. The reference group was PDUS−PDTS−. The model included age at diagnosis, gender and antibody status as fixed effects. Model performance was assessed using leave-one-out (loo) cross-validation, implemented using the loo package with results reported as posterior estimates (PEs) with 95% credible intervals (CrIs). This CrI threshold indicates a 0.95 probability that the true parameter lies within this range.

Descriptive statistics were used to report on the membership of, and the transition between, the concordance and discordance disease activity states.

Sensitivity analyses for change in disease activity states was conducted using the Simplified Disease Activity Index (SDAI) as a stringent measure for clinical disease activity assessment. SDAI ≤ 3.3 was used to define stringent clinical remission.

## Results

### Baseline stratification of early RA cohort according to presence of PDUS and/or PDTS

At the time of diagnosis with DAS (28-joints) with ESR (DAS28-ESR)  ≥ 3.2, a trial eligibility criterion, 63/120 (52%) were PDUS+PDTS+, 18/120 (15%) were PDUS+PDTS−, 19/120 (16%) were PDUS−PDTS+ and 20/120 (17%) were PDUS−PDTS−. Table 1 details the demographic, clinical and disease activity data.

**Table 1.** Baseline clinical and other imaging characteristics stratified by presence of PDUS and/or PDTS [continuous variables reported as median (IQR) and categorical variables reported as *n*/*N* (%)]

Characteristic	Overall, *N* = 120	PDUS+PDTS+, *N* = 63 (52%)	**PDUS+PDTS**−**, *N* = 18 (15%)**	**PDUS**−**PDTS+, *N* = 19 (16%)**	**PDUS**−**PDTS**−**, *N* = 20 (17%)**	*P*-value
Age (years) at diagnosis	52 (42, 61)	54(45, 62)	51 (46, 59)	44 (37, 53)	45 (38, 56)	**0.014**
Female gender	85/120 (71%)	42/63 (67%)	12/18 (67%)	14/19 (74%)	17/20 (85%)	0.4
Symptom duration (weeks) at diagnosis	20.28 (13.18, 30.75)	17.86 (12.43, 24.43)	26.72 (17.57, 39.14)	24.14 (18.36, 33.50)	26.72 (16.64, 33.61)	**0.023**
Early morning stiffness (min)	90 (30, 240)	110 (38, 270)	60(60, 226)	120(45, 210)	60 (5, 180)	0.4
Treatment group						0.2
MTX-TT	60/120 (50%)	26/60 (44%)	12/60 (20%)	11/60 (18%)	11/60 (18%)	
ETN** **+** **MTX	60/120 (50%)	37/60 (62%)	6/60 (10%)	8/60 (13%)	9/60 (15%)	
Seropositive (RF and/or ACPA) antibody status	106/120 (88%)	54/63 (86%)	16/18 (89%)	17/19 (89%)	19/20 (95%)	0.6
RF positive	87/120 (73%)	45/63 (71%)	14/18 (78%)	13/19 (68%)	15/20 (75%)	
ACPA positive	101/120 (84%)	53/63 (84%)	14/18 (78%)	17/19 (89%)	17/20 (85%)	
Tender joint count in 28 joints (TJC28)	11 (7, 17)	12(7, 19)	10 (7, 16)	10 (6, 16)	9 (5, 11)	0.15
Swollen joint count in 28 joints (SJC28)	5 (2, 9)	7 (4, 11)	4 (1, 8)	4 (2, 5)	2(1, 5)	**<0.001**
VAS (mm)—disease activity	58 (43, 74)	62 (45, 75)	60 (47, 70)	54 (38, 78)	53(37, 68)	0.7
ESR (mm/h)	32 (19, 50)	33 (21, 60)	33 (18, 65)	25 (18, 30)	28 (15, 46)	0.13
CRP (mg/l)	8 (2, 21)	11 (5, 28)	12 (6, 17)	4 (1,)	3 (1, 8)	**<0.001**
VAS (mm)—pain	59 (35, 71)	59 (43, 74)	54 (40, 68)	61 (34, 74)	44 (29, 67)	0.6
HAQ score	1.19 (0.86, 1.49)	1.19 (1.03, 1.45)	1.34 (0.73, 1.41)	1.26 (0.94, 1.49)	1.07 (0.44, 1.51)	0.6
DAS28-ESR	5.64 (4.88, 6.31)	6.12 (5.08, 6.81)	5.41 (5.09, 6.21)	5.31 (4.86, 5.64)	4.86 (4.21, 5.77)	**0.002**
SDAI	29.28 (20.63, 41.35)	35.43 (22.73, 45.97)	25.89 (21.91, 31.98)	26.30 (18.43, 34.33)	22.59 (13.69, 30.62)	**0.003**
Other imaging characteristics						
GS present	107/120 (89%)	63/63 (100%)	18/18 (100%)	14/19 (74%)	12/20 (60%)	**<0.001**
Erosions present	16/120 (13%)	14/63 (22%)	2/18 (11%)	0/19 (0%)	0/20 (0%)	**0.010**
Osteophytes present	26/120 (22%)	17/63 (27%)	6/18 (33%)	0/19 (0%)	3/20 (15%)	**0.021**

Bold font highlights significant results. PDUS+PDTS+: PDUS > 0 and PDTS > 0**;** PDUS+PDTS−: PDUS > 0 and PDTS = 0; PDUS−PDTS+: PDUS = 0 and PDTS > 0; PDUS−PDTS−: PDUS = 0 and PDTS = 0; PDUS: power Doppler US joint synovitis; PDTS: power Doppler tenosynovitis; MTX-TT: MTX (treat-to-target); ETN** **+** **MTX: etanercept** **+** **MTX; VAS: visual analogue score; DAS28-ESR: DAS (28-joints) with ESR; SDAI: Simplified Disease Activity Index; GS: Greyscale.

GS was present in all patients with PDUS+ (PDUS+PDTS+ and PDUS+PDTS− groups) and observed in 14/19 (74%) and 12/20 (60%) of PDUS−PDTS+ and PDUS−PDTS− groups, respectively. The PDUS+PDTS+ group had significantly shorter symptom duration (median 17.86 weeks compared with a median of 25 weeks or over in the other three groups) and the most active disease (*P* = 0.023); with the PDUS−PDTS− group having lower clinical disease activity scores (median DAS28-ESR = 4.86, SDAI = 22.59), swollen joint counts (median SJC28 = 2) and CRP (median = 2.66) than PDUS+PDTS+ (median DAS28-ESR = 6.12, SDAI = 35.43, SJC28 = 7, CRP = 11.28). The two groups with PDTS+ (PDUS+PDTS+ and PDUS−PDTS+) had numerically higher visual analogue score (VAS) pain (median 59 and 61, respectively). Erosions were only noted in patients with PDUS+, with higher proportions observed in the PDUS+PDTS+ group (22%) *vs* PDUS+PDTS− group (11%).

### Association of baseline PDUS and/or PDTS with subsequent remission

**Figure 1.**
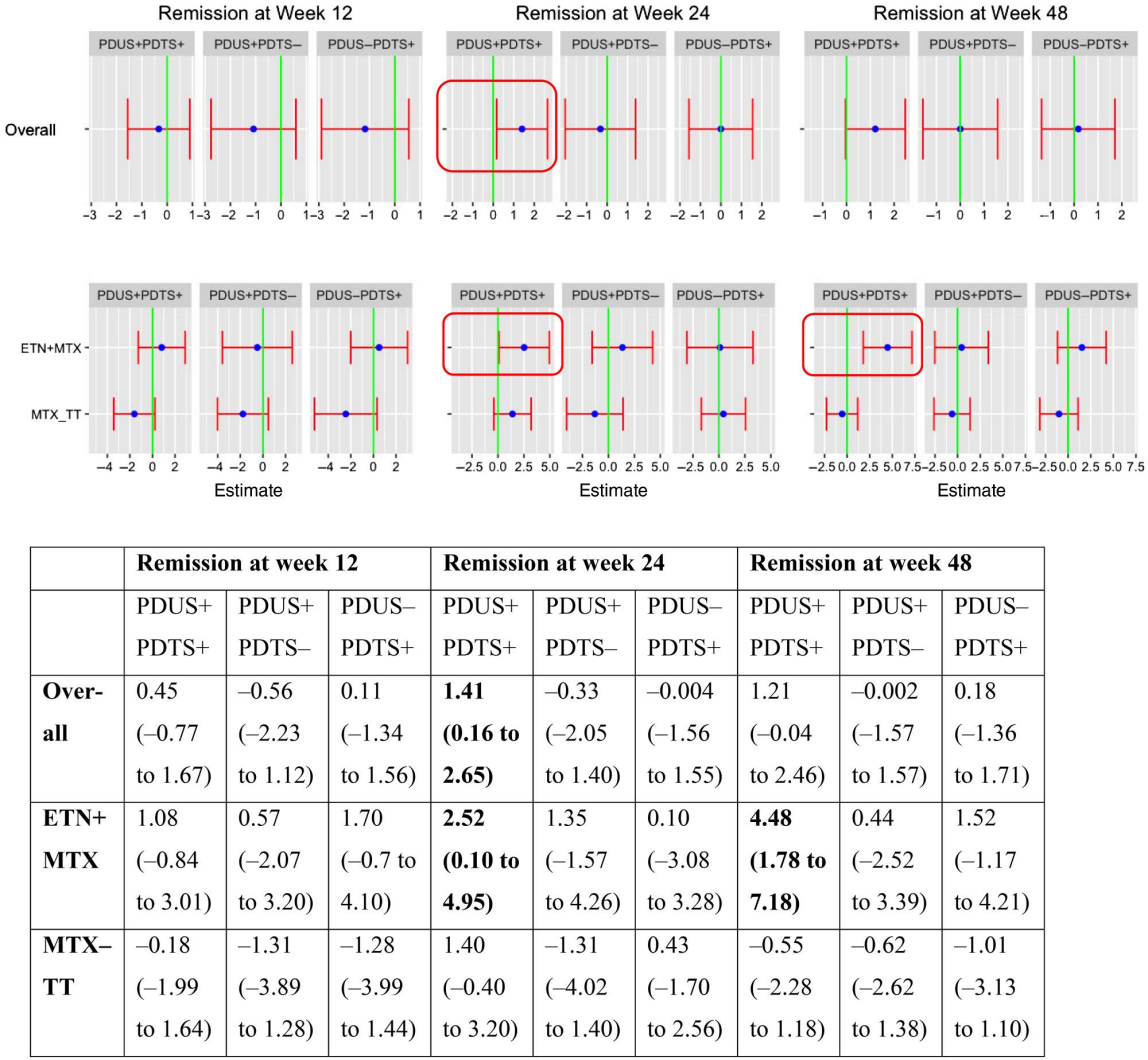
Association between the presence of baseline PDUS and/or PDTS with DAS28-ESR remission. PDUS and/or PDTS categorized as PDUS+PDTS+, PDUS+PDTS−, PDUS−PDTS+, PDUS−PDTS−. PDUS+PDTS+: PDUS > 0 and PDTS > 0; PDUS+PDTS−: PDUS > 0 and PDTS = 0; PDUS−PDTS+: PDUS = 0 and PDTS > 0; PDUS−PDTS−: PDUS = 0 and PDTS = 0; PDTS+ groups (with PDUS−PDTS− as the comparator group) and outcome of DAS28-ESR remission at weeks 12 (left), 24 (middle) and 48 (right) for the overall cohort (row 1) and stratified by randomized treatment strategy arm (row 2). Overall (row 1) and treatment strategy groupwise (row 2) posterior estimates are shown with 95% credible intervals. The zero estimate is indicated by a vertical line—if the error bars cross zero, then the result is not significant. Statistically significant results are highlighted with a box in the image and bold in the table below it. PDUS: power Doppler US joint synovitis; PDTS: power Doppler tenosynovitis; MTX-TT: MTX (treat-to-target); ETN** **+** **MTX: etanercept** **+** **MTX

Using PDUS−PDTS− as the reference group (*n*/*N* = 20/120), baseline PDUS+PDTS+ (*n*/*N* = 63/120) was associated with DAS28-ESR remission at week 24 (PE = 1.41, CrI = 0.16–2.65) (Fig. 1). This effect was observed in the same direction at week 48 but did not reach statistical significance (PE = 1.21, CrI = −0.04–2.46).

In the ETN + MTX group, 37/60 (62%) were PDUS+PDTS+ compared with 26/60 (44%) in the MTX-TT group (Table 1) at baseline. Within each treatment group, we analysed whether baseline PDUS and/or PDTS was also associated with subsequent remission. In the ETN + MTX group, there was a significant association of baseline PDUS+PDTS+ with DAS28-ESR remission at weeks 24 (PE = 2.52, CrI = 0.1–4.95) and 48 (PE = 4.48, CrI = 1.78–7.18), with no such association observed in the MTX-TT group. This association in the ETN + MTX group was also noted for SDAI remission at week 24 (PE = 4.88, CrI = 0.84–8.94) and 48 (PE = 2.78, CrI = 0.39–5.17) (Supplementary Fig. S1, available at *Rheumatology* online).

### Transition in concordance and discordance states over time

Next, we evaluated concordance and discordant states using DAS28-ESR and PDUS. In line with trial eligibility, all patients had active disease (moderate or high DAS28-ESR) at baseline of which 81/120 (68%) were DAS+PDUS+ and 39/120 (32%) were DAS+PDUS− (Table 2). Compared with DAS+PDUS− patients, DAS+PDUS+ patients were older (median age 53 *vs* 44), had higher DAS28-ESR (5.90 *vs* 5.16) and higher CRP (12 mg/l *vs* 4 mg/l), with a greater proportion in this group with GS (100% *vs* 67%, respectively), PDTS (78% *vs* 49%, respectively) and erosions (20% *vs* 0, respectively) (all *P* < 0.05).

**Table 2:** Baseline characteristics according to concordance/discordance between DAS28-ESR and PDUS

Characteristic	Overall, *N* = 120	DAS+PDUS+, *n*/*N* = 81/120 (68%)	DAS+PDUS−, *n*/*N* = 39/120 (32%)
Age at baseline (years)	52 (42, 61)	53 (45, 62)	44 (37, 55)
Female gender	85/120 (71%)	54/81 (67%)	31/39 (79%)
Symptom duration (weeks)	20.28 (13.18, 30.75)	19.00 (12.57, 27.14)	24.86 (17.22, 34.07)
Early morning stiffness (min)	90 (30, 240)	90 (45, 240)	79 (20, 180)
Treatment group			
MTX-TT	60/120 (50%)	38/81 (47%)	22/39 (56%)
ETN** **+** **MTX	60/120 (50%)	43/81 (53%)	17/39 (44%)
Seropositive antibody status	106/120 (88%)	70/81 (86%)	36/39 (92%)
RF positive	87/120 (73%)	59/81 (73%)	28/39 (72%)
ACPA positive	101/120 (84%)	67/81 (83%)	34/39 (87%)
Swollen joint count in 28 joints (SJC28)	5 (2, 9)	6 (3, 10)	3 (1, 5)
Tender joint count in 28 joints (TJC28)	11 (7, 17)	12 (7, 18)	9 (6, 14)
VAS—disease activity (mm)	58 (43, 74)	62 (46, 74)	54 (37, 74)
ESR (mm/h)	32 (19, 50)	33 (20, 62)	25 (18, 38)
CRP (mg/l)	8 (2, 21)	12 (5, 27)	4 (1, 9)
VAS—pain (mm)	59 (35, 71)	59 (41, 72)	55 (31, 70)
HAQ score	1.19 (0.86, 1.49)	1.19 (0.86, 1.41)	1.19 (0.68, 1.49)
DAS28-ESR	5.64 (4.88, 6.31)	5.90 (5.09, 6.67)	5.16 (4.62, 5.71)
SDAI	29.29 (20.63, 41.35)	31.50 (22.47, 44.49)	24.11 (17.15, 32.91)
Imaging features			
GS present	107/120 (89%)	81/81 (100%)	26/39 (67%)
Erosions present	16/120 (13%)	16/81 (20%)	0/39 (0%)
Osteophytes present	26/120 (22%)	23/81 (28%)	3/39 (8%)
PDUS+PDTS+	63/120 (52%)	63/81 (78%)	0/39 (0%)
PDUS+PDTS−	18/120 (15%)	18/81 (22%)	0/39 (0%)
PDUS−PDTS+	19/120 (16%)	0/81 (0%)	19/39 (49%)
PDUS−PDTS−	20/120 (17%)	0/81 (0%)	20/39 (51%)

All patients had active clinical disease (according to DAS28-ESR) as per trial recruitment criteria. DAS+PDUS+: DAS28-ESR > 2.6 and PDUS > 0; DAS+PDUS−: DAS28-ESR > 2.6 and PDUS = 0; PDUS: power Doppler US joint synovitis; PDTS: power Doppler tenosynovitis; MTX-TT: MTX (treat-to-target); ETN + MTX: etanercept + MTX; VAS: visual analogue score; DAS28-ESR: DAS (28-joints) with ESR; SDAI: Simplified Disease Activity Index; GS: Greyscale.

**Figure 2.**
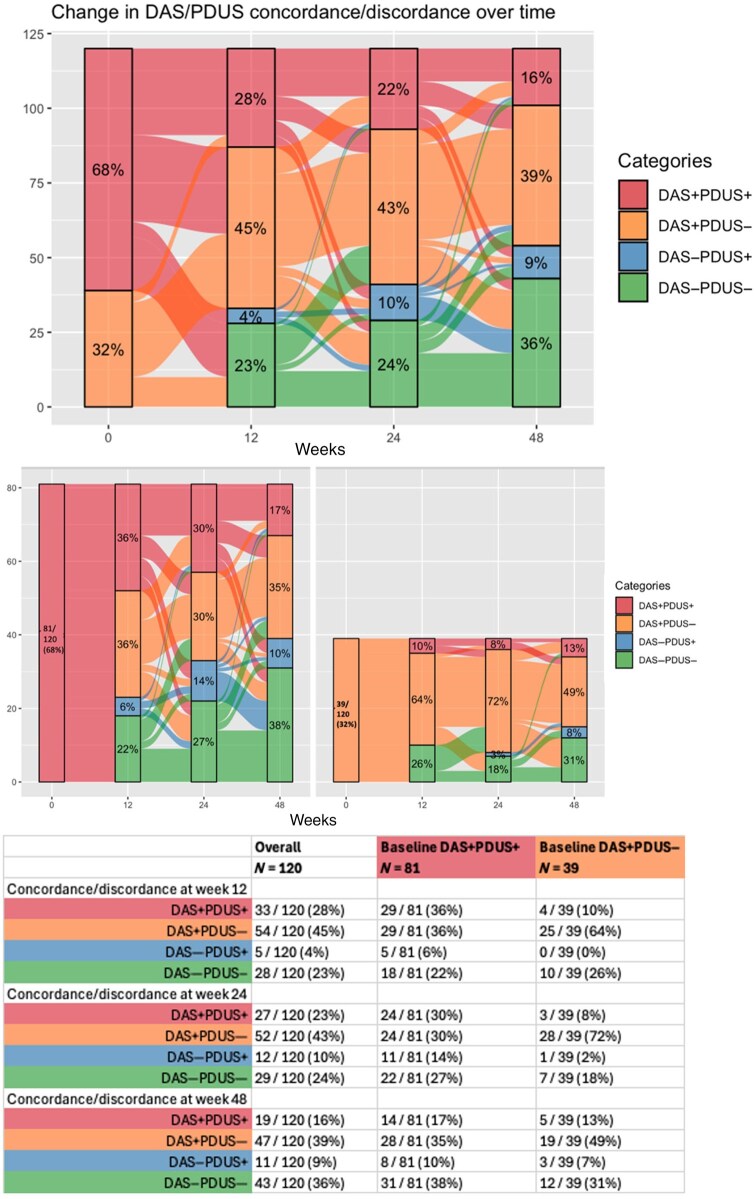
Longitudinal change in concordance/discordance states of DAS28-ESR and PDUS. DAS+PDUS+: DAS28-ESR** **>** **2.6 and PDUS** **>** **0; DAS+PDUS−** **=** **DAS28-ESR** **>** **2.6 and PDUS** **=** **0; DAS−PDUS+** **=** **DAS28-ESR** **≤** **2.6 and PDUS** **>** **0; DAS−PDUS−** **=** **DAS28-ESR** **≤** **2.6 and PDUS** **=** **0. Top image: Alluvial plot highlighting the transitions during the trial period—combined. Centre image: Alluvial plot faceted by baseline DAS+PDUS+ and DAS+PDUS− states. Lower table: week-wise transition states from baseline DAS+PDUS+ and DAS+PDUS− groups. PDUS: power Doppler joint synovitis

Fig. 2 illustrates the proportions in DAS+PDUS+ and DAS+PDUS− pre-treatment and change over time, detailing proportions in DAS+PDUS+, DAS+PDUS−, DAS−PDUS+ and DAS−PDUS− at each trial visit timepoint. This revealed an early (expected) transition of DAS+PDUS+ to DAS−PDUS− in 18/81 (22%) at week 12, with 29 (36%) remaining in DAS+PDUS+ but 29 (36%) moving to DAS+PDUS−. The proportion in DAS+PDUS− from baseline [39/120 (32%)] persisted at subsequent timepoints [54/120 (45%) at week 12, 52/120 (43%) at week 24 and 47/120 (39%) at week 48]. However, the individual membership of DAS+PDUS− changed over time—only 16/39 (41%) who were in this group at baseline remained as such through to week 48 (Supplementary Fig. S2, available at *Rheumatology* online). Of the 52 patients in DAS+PDUS− at week 24, 8 (15%) were in DAS+PDUS+ at week 12, 13 (25%) were in DAS−PDUS− at week 12. Of the 47 patients in DAS+PDUS− at week 48, 8 (17%) had been in DAS+PDUS+ at week 24 and 5 (11%) were in DAS−PDUS− at week 24 (Supplementary Fig. S2, available at *Rheumatology* online).

Concordance and discordance states between DAS28-ESR and PDTS were also analysed—82/120 (68%) were in DAS+PDTS+ and 38 (32%) were in DAS+PDTS− at baseline (Supplementary Table S1, available at *Rheumatology* online). Similar trends in transition were noted for PDTS (Supplementary Fig. S3, available at *Rheumatology* online).

These analyses were also performed using SDAI as the DAS. The results were comparable with those presented for DAS28-ESR (Supplementary Figs S4 and S5, available at *Rheumatology* online).

**Figure 3.**
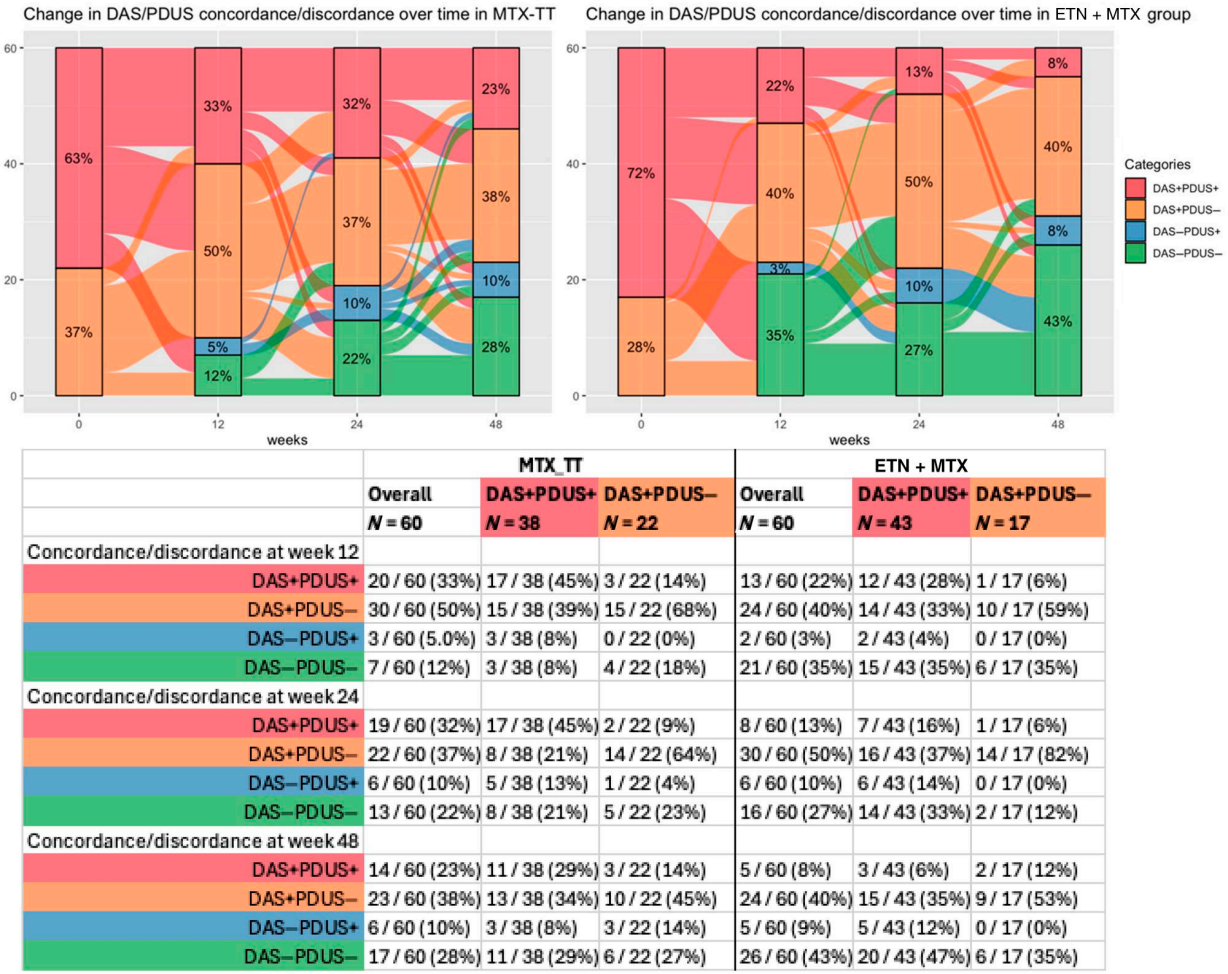
Longitudinal change in concordance/discordance states of DAS28-ESR and PDUS by treatment group. Left** **=** **MTX-TT group, Right** **=** **ETN+MTX group. DAS+PDUS+: DAS28-ESR** **>** **2.6 and PDUS** **>** **0; DAS+PDUS−: DAS28-ESR** **>** **2.6 and PDUS** **=** **0; DAS−PDUS+: DAS28-ESR** **≤** **2.6 and PDUS** **>** **0. Top image: Alluvial plot highlighting the transitions during the trial period. Lower table: week-wise transition states from baseline DAS+PDUS+ and DAS+PDUS− groups. PDUS: power Doppler joint synovitis; MTX-TT: MTX (treat-to-target); ETN** **+** **MTX: etanercept** **+** **MTX

Within treatment groups, only 7/60 (12%) individuals were DAS−PDUS− at week 12 in the MTX-TT group compared with 21/60 (35%) in the ETN + MTX group (Fig. 3). However, no significant differences were noted between treatment groups when evaluating with PDTS (Supplementary Fig. S6, available at *Rheumatology* online).

## Discussion

We report on novel post-hoc analyses of clinical disease activity state and PDUS and/or PDTS joint-complex inflammation in a treatment-naïve, early RA, randomized controlled-trial cohort. Our key findings are first, PDUS+PDTS+ was associated with remission, seen mainly in the ETN + MTX compared with MTX-TT group. Second, a third of patients at the time of diagnosis were in DAS+power Doppler US−, and while this proportion was maintained over time on a group level, individual shifts into and from DAS+power Doppler US− continued to occur longitudinally including into remission. Third, DAS−power Doppler US− remission emerged early in the ETN + MTX compared with the MTX-TT group.

There is extensive literature on clinical disease activity and associated imaging [12, 13, 19], but few studies have sought to phenotype the joint and tendon complex, its association with treatment response, and the concept of concordance/discordance and their transition over time. This treatment-naïve, early RA cohort was categorized into four subgroups based on the presence or absence of PDUS and PDTS, with the majority demonstrating joint and tendon power Doppler US. Those with PDTS alone had lower acute-phase response, joint GS presence and absence of erosions compared with groups with PDUS (PDUS+PDTS−, PDUS+PDTS+). However, this group had comparable tender and swollen joint counts to the group with PDUS alone. This is consistent with studies of ACPA+ at-risk RA cohorts that have identified the presence of tenosynovitis as a predictor of progression to RA [20–22] and an imaging biomarker in early RA that is associated with poor clinical [23] and radiographic outcomes [10, 21]. These data also highlight that recording of clinical joint swelling cannot differentiate between synovitis and tenosynovitis. In MRI studies, tenosynovitis has been shown to be associated with joint swelling and tenderness, and this association was independent of concurrent MSUS-detected synovitis [8]. We also observed that there was no significant difference in VAS—pain and HAQ scores between these groups. This suggests that pain and high functional disability are early features of RA disease, irrespective of the underlying imaging phenotype, and aligns with previous data showing poor association with power Doppler US [13, 24].

Presence of both joint synovitis and tenosynovitis at baseline was associated with subsequent clinical remission in the overall group, but within each treatment arm this only applied to the group randomized to first-line TNFi + MTX. No such association was observed in those treated with first-line MTX-TT. A possible explanation for our results may be the patient population—the VEDERA trial included people with early disease (median symptom duration < 21 weeks), rather than established cohorts. The presence of both synovitis and tenosynovitis is a marker of aggressive disease and hence may be more responsive to aggressive early treatment. While there has been significant interest in PDUS and PDTS as predictors of disease flare [23, 25], data on the prediction of remission are limited. The presence of PDUS has been associated with various imaging-based outcome measures, such as radiographic- [26], US- [27] and MRI-detected erosions [28]. Concurrent with these findings, US erosions were mainly a feature of the PDUS group (with or without PDTS) in our study.

Discrepancy between measured disease activity and MSUS-determined synovitis [29–31] and, to a lesser extent, tenosynovitis [32, 33] is well recognized. We categorized patients as DAS+PDUS+, DAS+PDUS−, DAS−PDUS+ and DAS−PDUS− states and identified a third of patients classifiable for RA as discordant (DAS+PDUS−) at the time of diagnosis. The overall proportion of DAS+PDUS− participants remained largely unchanged, but the individual membership was dynamic. This suggests discordant measures, typically attributed to chronic pain states, appear to be modifiable with DMARDs in a proportion of cases. This was seen in both MTX-TT and ETN + MTX groups. This discordance was first reported by Horton and colleagues and in more established RA [34]. Such observations have led to the development of measures that solely reflect local joint level inflammation (eg, 2-component-DAS28) [35]. However, composite indices originally emerged in recognition of RA being a systemic disease, with patient-reported outcomes being one of the most sensitive to change among all RA core set measures [36]. Comparative trial data of biologic DMARDs and janus-kinase (JAK) inhibitor targeted synthetic DMARDs suggest the latter may confer effects on pain and physical function over and above that associated with disease activity (inflammation) suppression [37, 38]. It, therefore, remains important to not dismiss wider indicators of activity, as has been previously emphasized [39], and/or attribute specific disease assessment components to mechanisms that have not been verified. Indeed, in this study we did not detect any significant differences in patient-reported VAS and tender joint count when stratifying by joint-complex inflammation. There was also early achievement of concordant DAS28-ESR and PDUS remission noted in the ETN + MTX arm, suggesting that aggressive treatment may be more successful in enabling joint-complex remission in early RA.

The findings from this study underscore the complexities of assessing and managing RA, particularly in the context of clinical trials and patient selection. Misclassification of the PDUS−PDTS− subgroup as RA at baseline was unlikely, given the eligibility criteria for the ‘VEDERA’ trial, which required moderate-to-severe disease activity, and the fact that all patients met the 2010 ACR/EULAR classification criteria for RA. While the baseline presence of joint synovitis appears to be associated with subsequent remission, the other key finding was that those with raised clinical disease activity without such power Doppler US (that we often regard as not amenable to DMARD intervention) in fact displayed change in the DAS28 and/or power Doppler US traits, including remission state. These observations highlight the risks of relying heavily on isolated imaging results and/or patient-reported symptoms. We would suggest that there is still limited understanding of such clinical-imaging phenotypes such that excluding certain subgroups (eg, those without clear MSUS activity) from trials may be premature.

Our study has some key limitations. This was a post-hoc analysis of a randomized controlled-trial (RCT) cohort that needs validation with a dedicated study to confirm these preliminary findings. In addition, the MSUS assessment comprised a limited number of joints that may have missed actively inflamed joints. However, dominant and/or symptomatic joints typically underlie the majority of active disease, especially in early RA [40, 41], supporting the validity of the approach. Also, limited MSUS captures real-world assessment, providing a pragmatic approach that can be translated into clinical practice. The influence of stable NSAID use and protocol-permitted steroid administration [42] on power Doppler US+ status may also be potential confounders that would be challenging to eliminate. We could also have defined controlled disease as achieving low clinical disease activity, but this would not have been in keeping with the ideal target for a very early RA cohort. Analysing with this definition, the results remained largely unchanged, with numerical differences in the concordance/discordance group membership but overall maintenance of the DAS+power Doppler US− group over time (data not shown). Similarly, we considered a total power Doppler US score of ≥1 as evidence of active synovitis/tenosynovitis. Various studies have highlighted the clinical importance of mild power Doppler US [12, 43, 44], and it was deemed important not to miss any evidence of joint or tendon inflammation, especially in a very early RA cohort and with a limited MSUS assessment. We also did not check FM scores as part of the study. Our data showed the presence of local joint tenderness in the absence of synovitis or tenosynovitis at baseline, indicating the symptoms may not necessarily be attributable to FM trigger points and were likely related to disease-related processes. This aligns with observations during the development phase of ‘at-risk’ RA, when joint pain is often an index symptom that precedes the development of inflammation [45, 46]. In addition, data on the pathogenicity of autoantibodies such as ACPA and RF and their association with arthralgia [47, 48] imply local joint tenderness here may be related to disease development rather than being a non-specific symptom.

Finally, it is unclear whether the findings from this study, which focuses on very early disease, are applicable across the disease continuum. Study of established RA cohorts is needed to confirm this. Studies with more comprehensive MSUS to acknowledge the more varied joint involvement that may be observed in later stages of RA may also be needed.

In summary, this study reports that in new-onset, treatment-naïve RA, the presence of joint-complex power Doppler US at baseline is associated with subsequent remission. DAS+power Doppler US− emerges early, but like DAS+power Doppler US+ and remission, is a dynamic state, indicating opportunity for therapeutic targeting. Validating these observations and understanding the basis for these states could inform more effective stratification and the development of personalized treatment strategies.

The National Research Ethics Service [Leeds (West) Research Ethics Committee] approved the protocol (reference 10/H1307/138) and its amendments. All participants consented to the VEDERA trial (10/H1307/138). The study was conducted in accordance with the principles of the Declaration of Helsinki.

## Supplementary Material

keaf098_Supplementary_Data

## Data Availability

All data relevant to the study are included in the article or uploaded as online supplementary information. Additional data are available on reasonable request.
