# Supplementary material for: Baseline synovitis–tenosynovitis is associated with remission in early rheumatoid arthritis, but discordance with disease activity is a changeable state
Source: Rheumatology (Oxford). 2025 Feb 17;64(6):3468–77. doi: 10.1093/rheumatology/keaf098 (PMC12107076; doi:10.1093/rheumatology/keaf098)
Supplement: keaf098_Supplementary_Data [file keaf098_supplementary_data.docx]

**RHE-24-2656: Baseline synovitis-tenosynovitis associates with remission in early rheumatoid arthritis but discordance with disease activity is a changeable state**

**
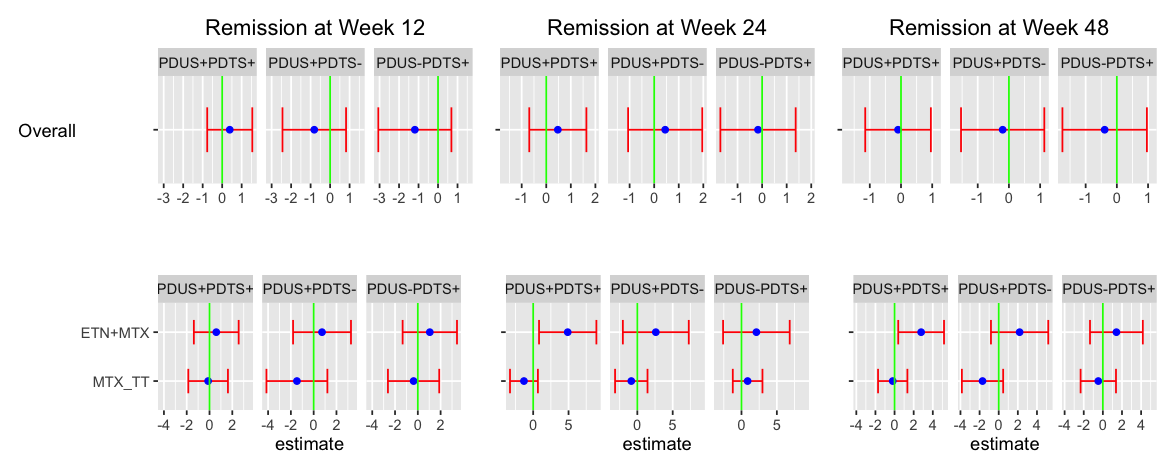
Supplementary figures and tables**

|  | **Remission at week 12** | | | **Remission at week 24** | | | **Remission at week 48** | | |
| --- | --- | --- | --- | --- | --- | --- | --- | --- | --- |
|  | PDUS+  PDTS+ | PDUS+  PDTS- | PDUS-PDTS+ | PDUS+  PDTS+ | PDUS+  PDTS- | PDUS-PDTS+ | PDUS+  PDTS+ | PDUS+  PDTS- | PDUS-PDTS+ |
| **Over-all** | 0.19  (-1.02 to 1.39) | -0.63  (-2.36 to 1.09) | 0.15  (-1.33 to 1.62) | 0.84  (0.42 to 2.11) | -0.38  (-2.08 to 1.33) | 0.67  (-0.82 to 2.16) | 0.89  (-0.30 to 2.08) | -0.14  (-1.57 to 1.30) | 0.31  (-1.10 to 1.71) |
| **ETN+MTX** | 0.59  (-1.37 to 2.56) | 0.73  (-1.81 to 3.27) | 1.05  (-1.33 to 3.44) | **4.88**  **(0.83 to 8.94)** | 2.61  (-2.05 to 7.27) | 2.11  (-2.60 to 6.82) | **2.78**  **(0.39 to 5.17)** | 2.19  (-0.81 to 5.18) | 1.42  (-1.34 to 4.18) |
| **MTX-TT** | -0.12  (-1.86 to 1.62) | -1.47  (-4.14 to 1.20) | -0.37  (-2.62 to 1.88) | -1.30  (-3.27 to 0.67) | -0.85  (-3.15 to 1.44) | 0.87  (-1.23 to 2.97) | -0.19  (-1.73 to 1.34) | -1.69  (-3.85 to 0.47) | -0.48  (-2.33 to 1.37) |

Figure S1: Association between the presence of baseline PDUS and/or PDTS, categorised as PDUS+PDTS+, PDUS+PDTS- or PDUS-PDTS+ groups (with PDUS-PDTS- as the comparator group) and outcome of SDAI remission at weeks 12 (left), 24 (middle) and 48 (right) for the overall cohort (row 1) and stratified by randomised treatment strategy arm (row 2). Overall (row 1) and treatment strategy groupwise (row 2) posterior estimates are shown with 95% credible intervals. The zero estimate is highlighted by a green line – if the error bars cross zero then the result is not significant. Statistically significant results highlighted with a red box in image and bold in table underneath.

PDUS = power Doppler joint synovitis, PDTS = power Doppler tenosynovitis, MTX-TT = Methotrexate (treat-to-target), ETN + MTX = Etanercept + Methotrexate

| **Characteristic** | **Overall, N = 120** | **DAS+PDTS+, N = 82 (68%)** | **DAS+PDTS-, N = 38 (32%)** |
| --- | --- | --- | --- |
| Age at baseline (yrs) | 52 (42, 61) | 53 (44, 61) | 48 (39, 57) |
| Female gender | 85 / 120 (71%) | 56 / 82 (68%) | 29 / 38 (76%) |
| Symptom duration (weeks) | 20.28 (13.18, 30.75) | 19.07 (12.86, 27.61) | 26.72 (16.90, 36.18) |
| Early morning stiffness (mins) | 90 (30, 240) | 115 (34, 240) | 60 (19, 182) |
| Treatment group |  |  |  |
| MTX_TT | 60 / 120 (50%) | 37 / 82 (45%) | 23 / 38 (61%) |
| ETN+MTX | 60 / 120 (50%) | 45 / 82 (55%) | 15 / 38 (39%) |
| Seropositive antibody status | 106/120 (88%) | 71/82 (87%) | 35/38 (92%) |
| RF positive | 87/120 (73%) | 58/82 (71%) | 29/38 (76%) |
| ACPA positive | 101/120 (84%) | 70/82 (85%) | 31/38 (82%) |
| Swollen joints (SJC28) | 5 (2, 9) | 6 (3, 9) | 3(1, 6) |
| Tender joints (TJC28) | 11 (7, 17) | 12 (7, 18) | 9(6, 14) |
| VAS – Disease activity (mm) | 58.00 (43.00, 74.00) | 61.00 (43.25, 75.00) | 55.50 (43.50, 68.00) |
| Erythrocyte sedimentation rate (mm/hr) | 31.51 (18.69, 50.41) | 30.42 (20.09, 50.94) | 32.14 (15.51, 49.90) |
| C-reactive protein | 8.37 (2.22, 21.25) | 9.67 (3.25, 25.27) | 6.36 (1.37, 15.46) |
| VAS – pain (mm) | 58.50 (35.00, 71.00) | 59.50 (40.00, 74.00) | 49.50 (29.75, 68.00) |
| HAQ score | 1.19 (0.86, 1.49) | 1.19 (0.96, 1.49) | 1.15 (0.57, 1.47) |
| DAS28-ESR | 5.64 (4.88, 6.31) | 5.86 (4.98, 6.60) | 5.24 (4.68, 6.01) |
| SDAI |  |  |  |
| Imaging features |  |  |  |
| GS present | 107 / 120 (89%) | 77 / 82 (94%) | 30 / 38 (79%) |
| Erosions present | 16 / 120 (13%) | 14 / 82 (17%) | 2 / 38 (5.3%) |
| Osteophytes present | 26 / 120 (22%) | 17 / 82 (21%) | 9 / 38 (24%) |
| PDUS+PDTS+ | 63 / 120 (52%) | 63 / 82 (77%) | 0 / 38 (0%) |
| PDUS+PDTS- | 18 / 120 (15%) | 0 / 82 (0%) | 18 / 38 (47%) |
| PDUS-PDTS+ | 19 / 120 (16%) | 19 / 82 (23%) | 0 / 38 (0%) |
| PDUS-PDTS- | 20 / 120 (17%) | 0 / 82 (0%) | 20 / 38 (53%) |

Table S1: Baseline characteristics according to concordance/discordance between DAS28-ESR and PDTS. All patients had active disease (according to DAS28-ESR) at baseline as per trial recruitment. DAS+PDTS+ = DAS28-ESR > 2.6 and PDTS > 0, DAS+PDTS- = DAS28-ESR > 2.6 and PDTS = 0

PDUS = power Doppler joint synovitis, PDTS = power Doppler tenosynovitis, MTX-TT = Methotrexate (treat-to-target), ETN + MTX = Etanercept + Methotrexate, RF = Rheumatoid factor, ACPA = Anti-citrullinated peptide antibody, VAS = visual analogue score, HAQ = Health Assessment Questionnaire, DAS28-ESR = Disease activity score (28-joints) with erythrocyte sedimentation rate, SDAI = Simplified Disease Activity Index, GS = Greyscale.


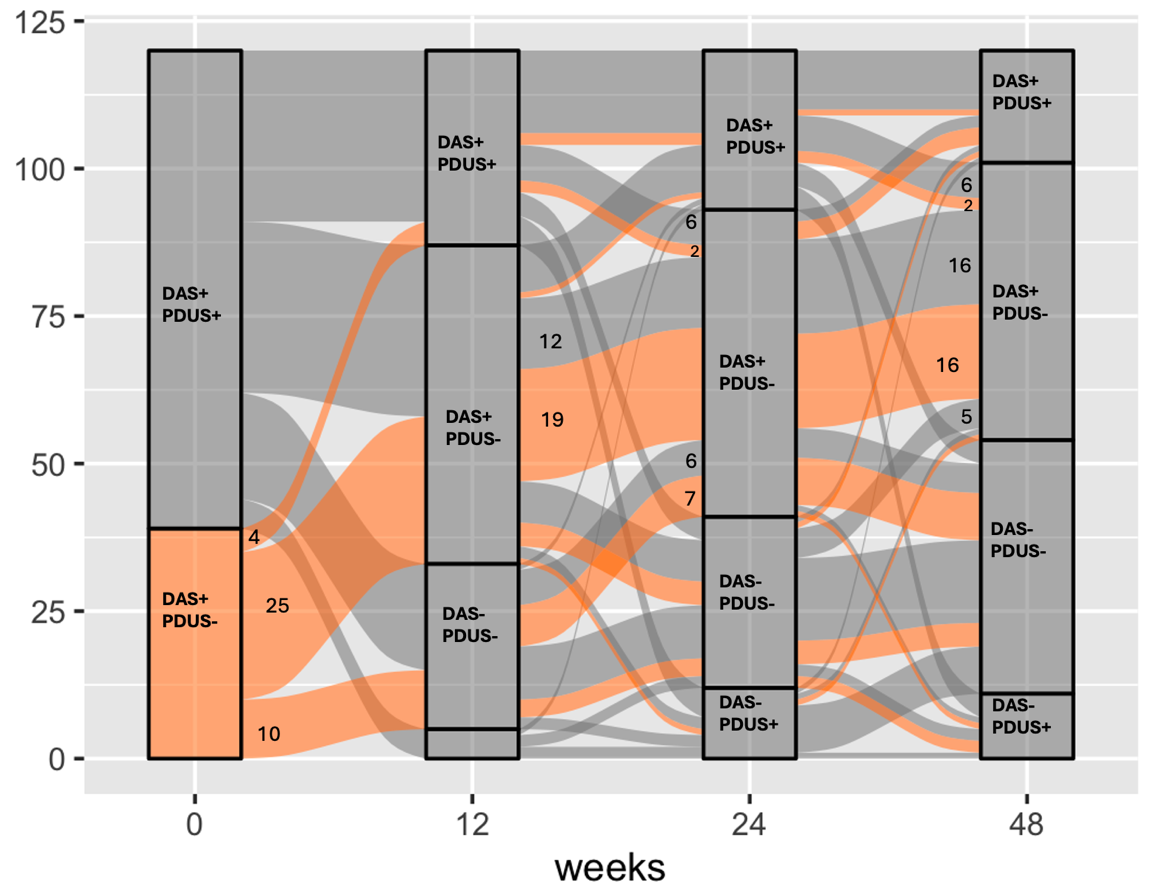


|  | **Week 24 DAS+PDUS+** | **Week 24 DAS+PDUS-** | **Week 24**  **DAS-PDUS+** | **Week 24**  **DAS-PDUS-** |
| --- | --- | --- | --- | --- |
| **Wk12 Overall, N = 120** | 27 / 120 (23%) | 52 / 120 (43%) | 12 / 120 (10%) | 29 / 120 (24%) |
| **Wk12 DAS+PDUS+**  **N = 33 / 120 (28%)** | 16 / 33 (48%) | 8 / 33 (24%) | 5 / 33 (16%) | 4 / 33 (12%) |
| **Wk12 DAS+PDUS-**  **N = 54 / 120 (45%)** | 9 / 54 (17%) | 31 / 54 (57%) | 3 / 54 (6%) | 11 / 54 (20%) |
| **Wk12 DAS-PDUS+**  **N = 5 / 120 (4%)** | 1 / 5 (20%) | 0 / 5 (0%) | 2 / 5 (40%) | 2 / 5 (40%) |
| **Wk12 DAS-PDUS-**  **N = 28 / 120 (23%)** | 1 / 28 (4%) | 13 / 28 (46%) | 2 / 28 (7%) | 12 / 28 (43%) |

|  | **Week 48 DAS+PDUS+** | **Week 48 DAS+PDUS-** | **Week 48**  **DAS-PDUS+** | **Week 48**  **DAS-PDUS-** |
| --- | --- | --- | --- | --- |
| **Wk24 Overall, N = 120** | 19 / 120 (16%) | 47 / 120 (39%) | 11 / 120 (9%) | 43 / 120 (36%) |
| **Wk24 DAS+PDUS+**  **N = 27 / 120 (23%)** | 11 / 27 (40%) | 8 / 27 (30%) | 4 / 27 (15%) | 4 / 27 (15%) |
| **Wk24 DAS+PDUS-**  **N = 52 / 120 (43%)** | 5 / 52 (10%) | 32 / 52 (62%) | 2 / 52 (3%) | 13 / 52 (25%) |
| **Wk24 DAS-PDUS+**  **N = 12 / 120 (10%)** | 1 / 12 (8%) | 2 / 12 (17%) | 1 / 12 (8%) | 8 / 12 (67%) |
| **Wk24 DAS-PDUS-**  **N = 29 / 120 (24%)** | 2 / 29 (7%) | 5 / 29 (17%) | 4 / 29 (14%) | 18 / 29 (62%) |

Figure S2: Top – Alluvial plot highlighting shifts into and out of DAS+PDUS- group. Middle – table of shift in concordance/discordance states from week 12 to week 24. Bottom – table of shift in concordance/discordance states from week 24 to week 48.

DAS+PDUS+ = DAS28-ESR > 2.6 and PDUS > 0

DAS+PDUS- = DAS28-ESR > 2.6 and PDUS = 0

DAS-PDUS+ = DAS28-ESR ≤ 2.6 and PDUS > 0

DAS-PDUS- = DAS28-ESR ≤ 2.6 and PDUS = 0

PDUS = power Doppler joint synovitis.


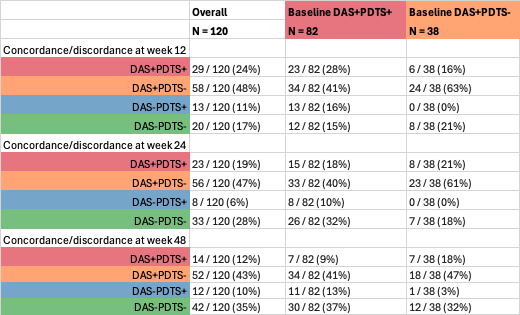

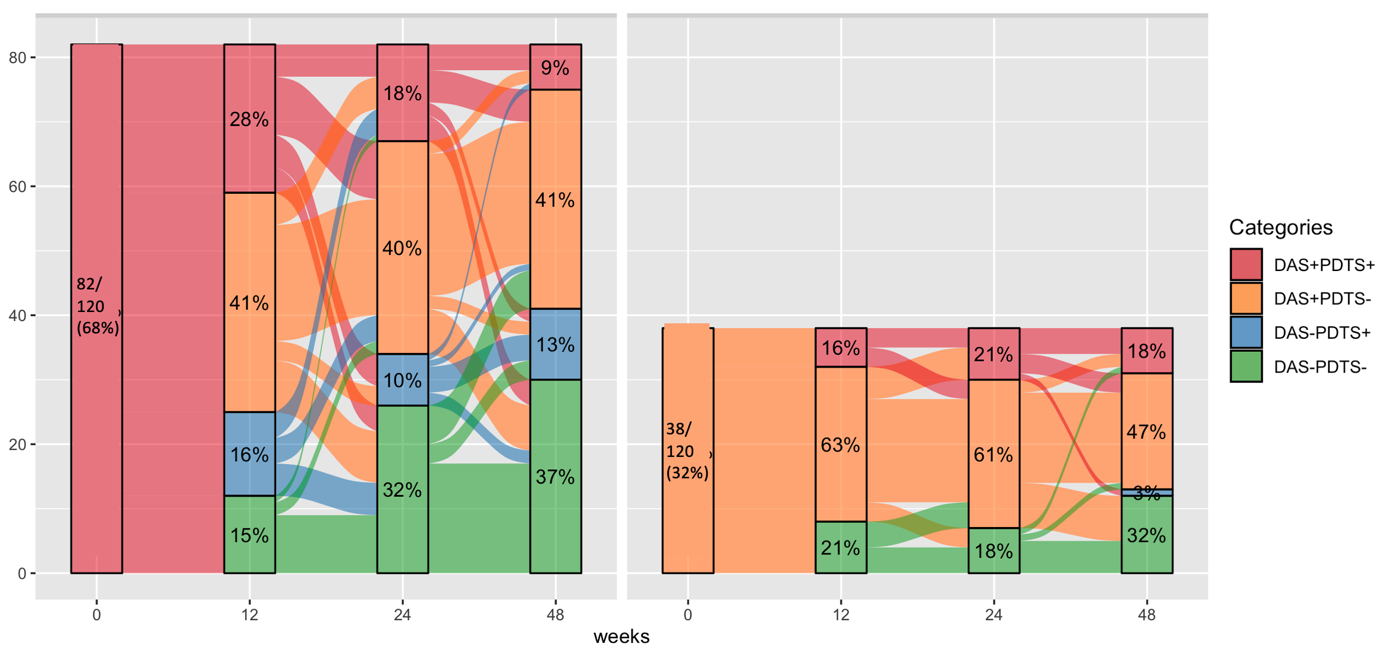

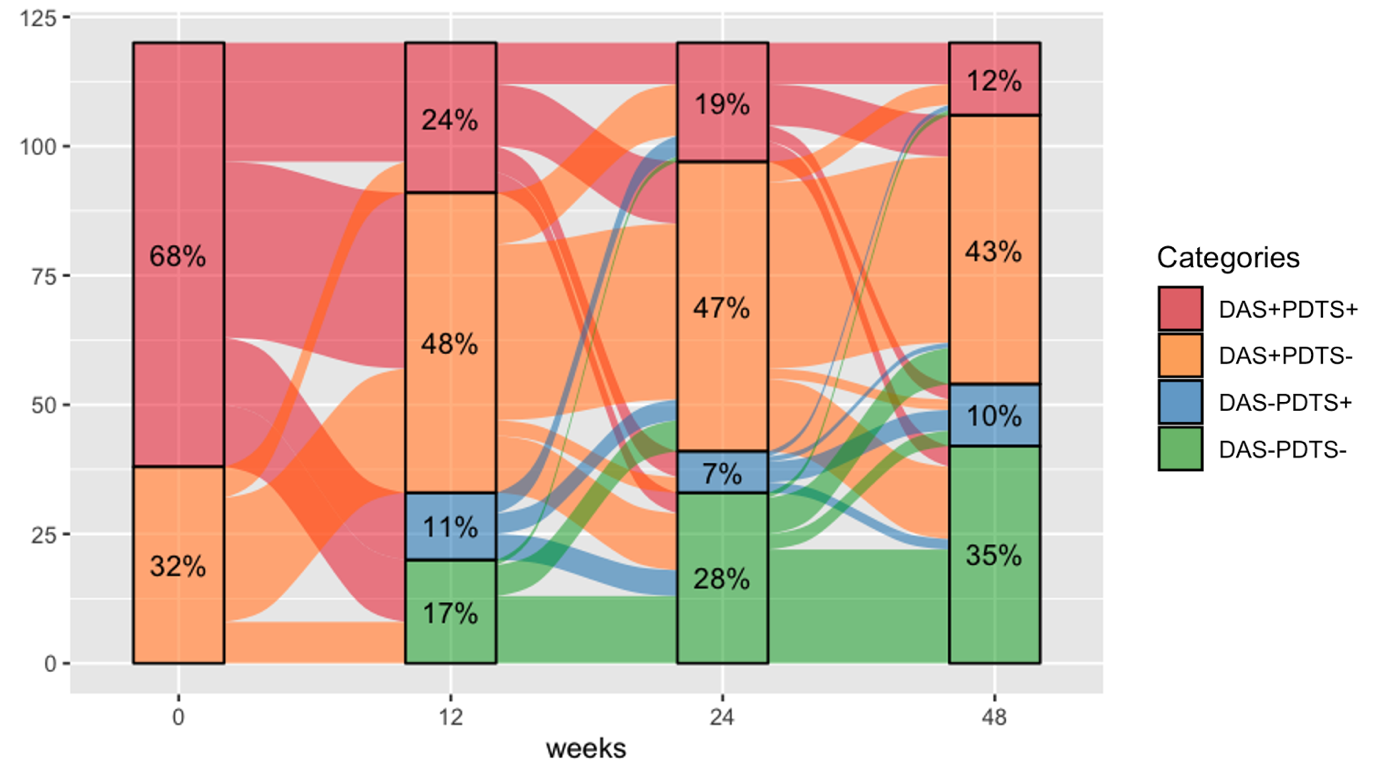


Figure S3: Longitudinal change in concordance/discordance states of DAS28-ESR and PDTS.

DAS+PDTS+ = DAS28-ESR > 2.6 and PDTS > 0

DAS+PDTS- = DAS28-ESR > 2.6 and PDTS = 0

DAS-PDTS+ = DAS28-ESR ≤ 2.6 and PDTS > 0

DAS-PDTS- = DAS28-ESR ≤ 2.6 and PDTS = 0

Top image: Alluvial plot highlighting the transitions during the trial period – combined

Middle image: Alluvial plot faceted by baseline DAS+PDTS+ and DAS+PDTS- states

Table: Week wise transition states from baseline DAS+PDTS+ and DAS+PDTS- groups.

PDTS = power Doppler tenosynovitis.


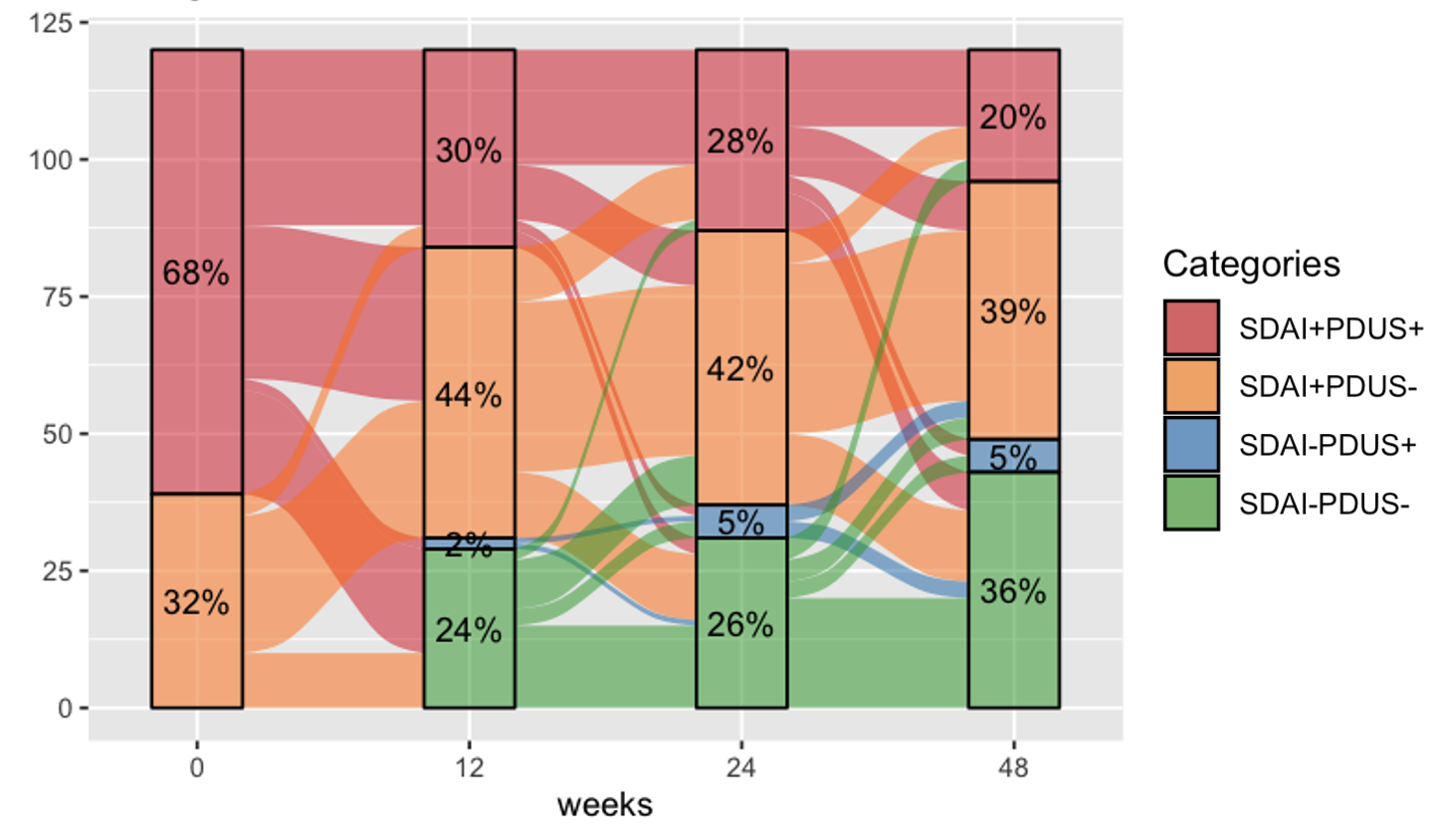

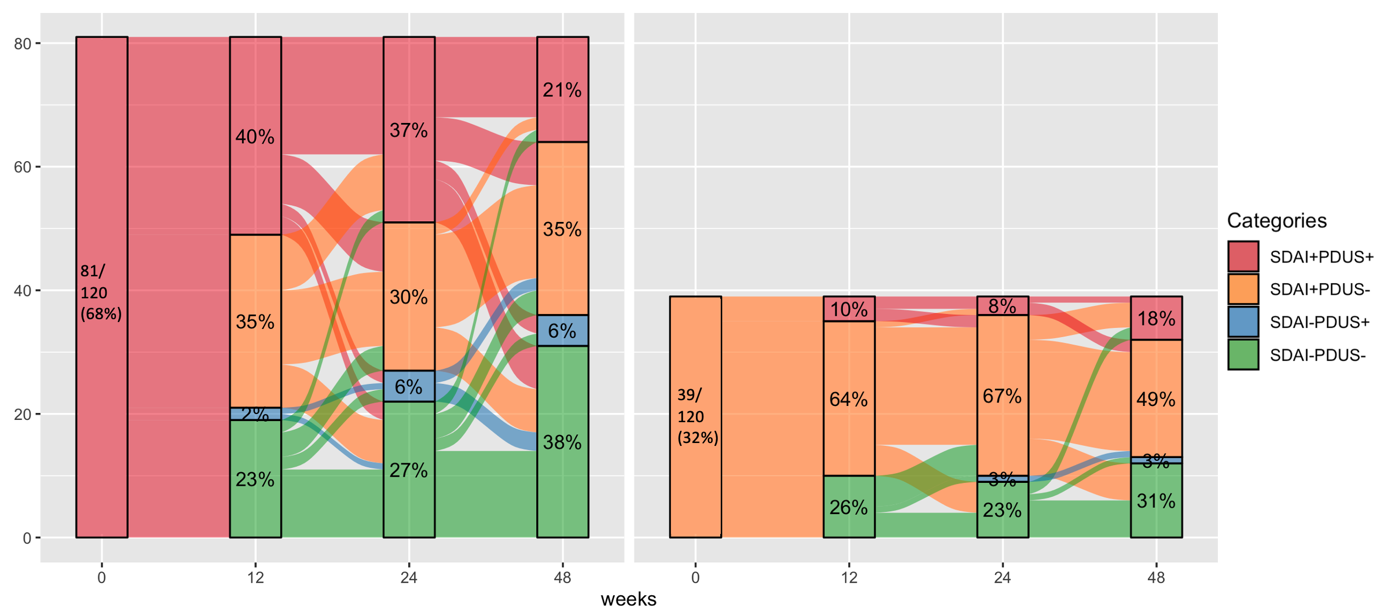


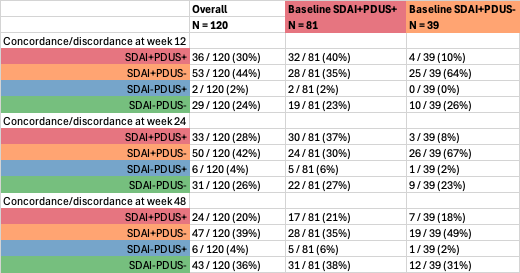


Figure S4: Longitudinal change in concordance/discordance states of SDAI and PDUS.

SDAI+PDUS+ = SDAI > 3.3 and PDUS > 0

SDAI+PDUS- = SDAI > 3.3 and PDUS = 0

SDAI-PDUS+ = SDAI ≤ 3.3 and PDUS > 0

SDAI-PDUS- = SDAI ≤ 3.3 and PDUS = 0

Top image: Alluvial plot highlighting the transitions during the trial period – combined

Middle image: Alluvial plot faceted by baseline SDAI+PDUS+ and SDAI+PDUS- states

Table: Week wise transition states from baseline SDAI+PDUS+ and SDAI+PDUS- groups.

SDAI = Simplified Disease Activity Index, PDUS = power Doppler joint synoviti


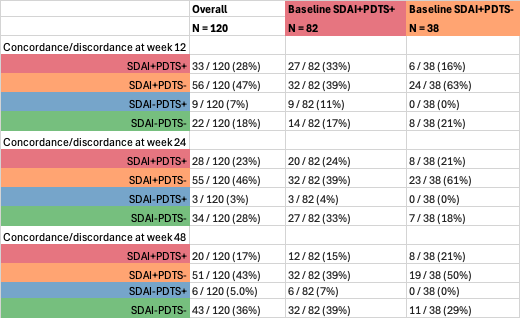

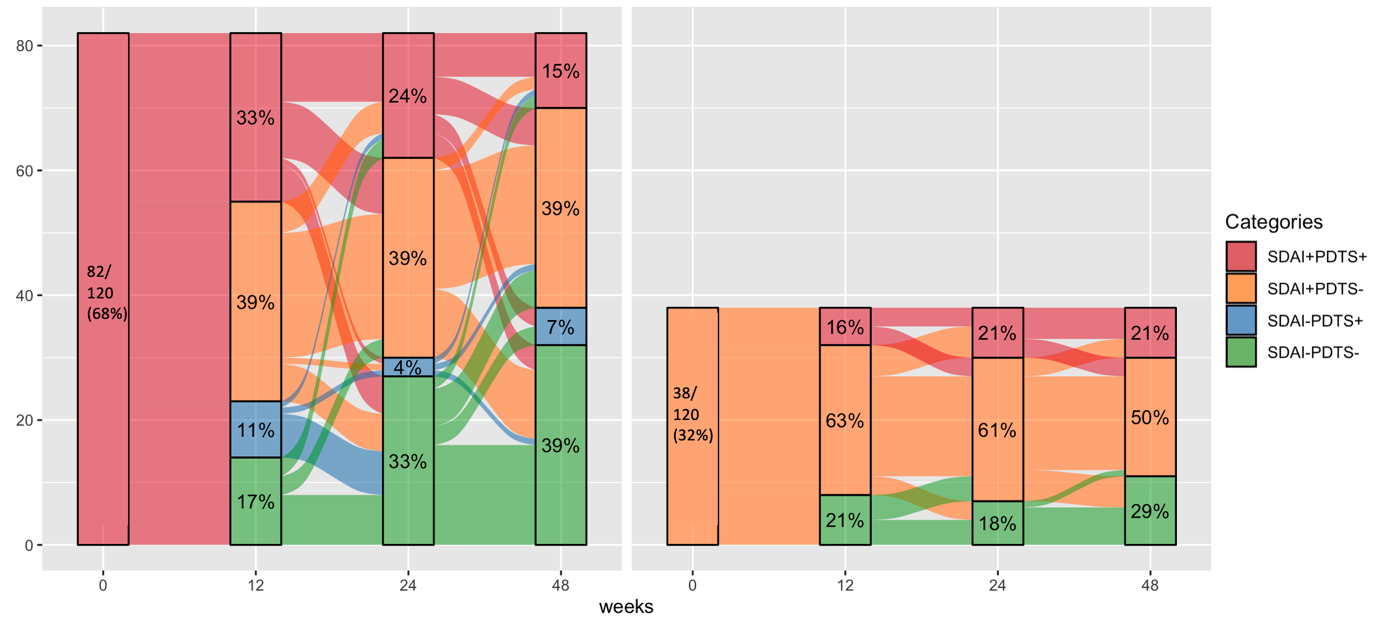

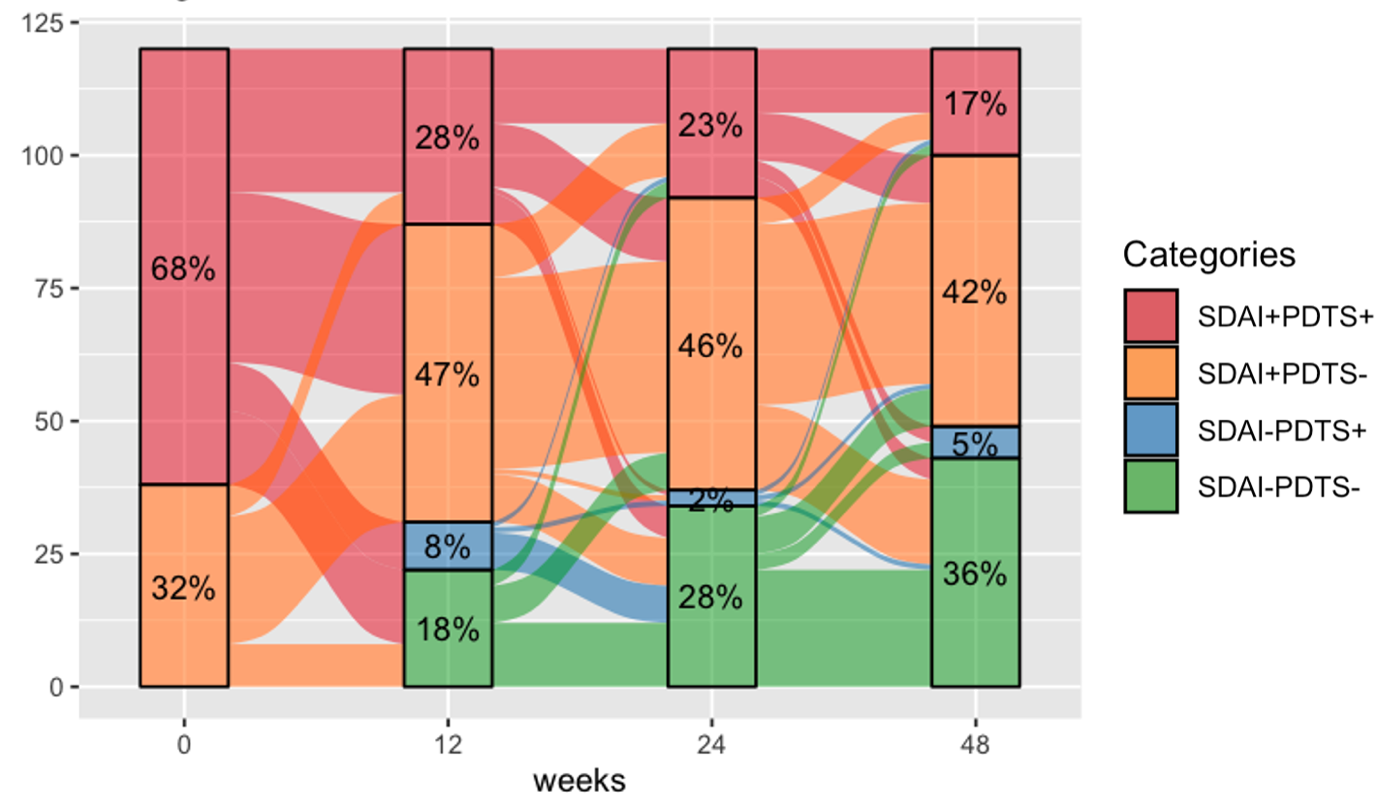


Figure S5: Longitudinal change in concordance/discordance states of SDAI and PDTS.

SDAI+PDTS+ = SDAI > 3.3 and PDTS > 0

SDAI+PDTS- = SDAI > 3.3 and PDTS = 0

SDAI-PDTS+ = SDAI ≤ 3.3 and PDTS > 0

SDAI-PDTS- = SDAI ≤ 3.3 and PDTS = 0

Top image: Alluvial plot highlighting the transitions during the trial period - – combined

Middle image: Alluvial plot faceted by baseline SDAI+PDTS+ and SDAI+PDTS- states

Table: Week wise transition states from baseline SDAI+PDTS+ and SDAI+PDTS- groups.

SDAI = Simplified Disease Activity Index, PDTS = power Doppler tenosynovitis.


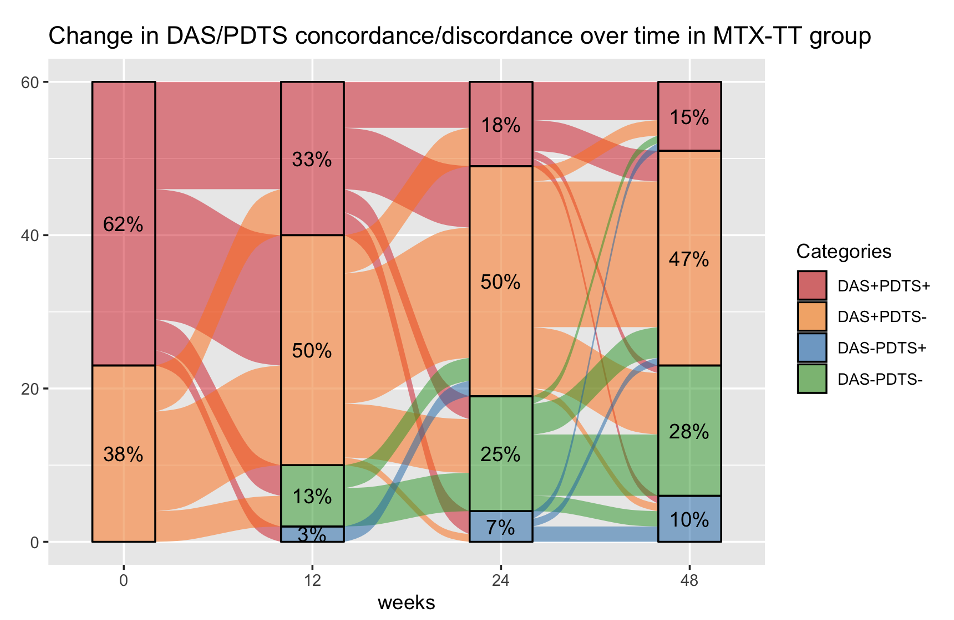


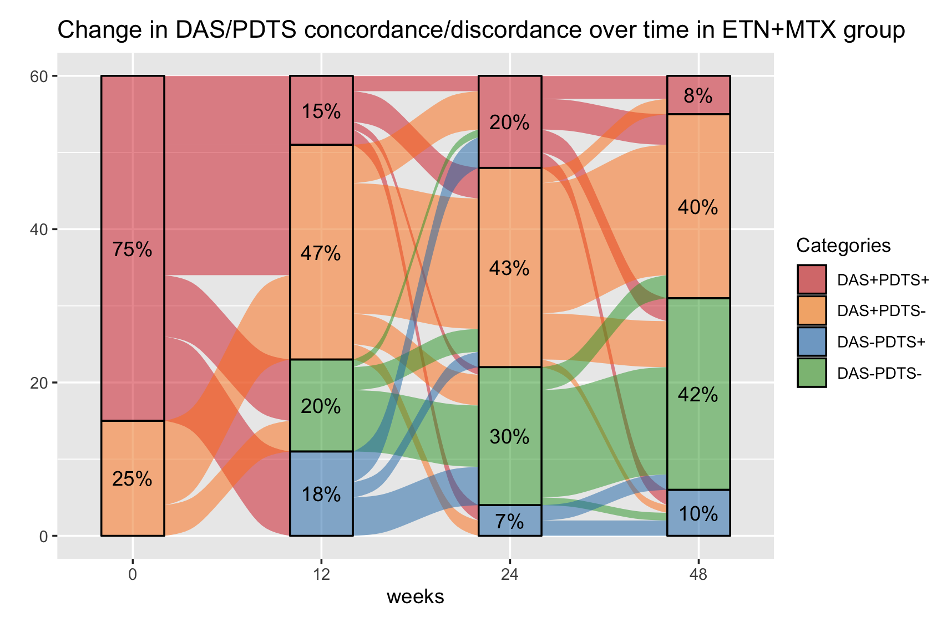


Figure S6: Longitudinal change in concordance/discordance states of DAS28-ESR and PDTS by treatment group. Top= MTX-TT group, Bottom = ETN+MTX group

DAS+PDTS+ = DAS28-ESR > 2.6 and PDTS > 0

DAS+PDTS- = DAS28-ESR > 2.6 and PDTS = 0

DAS-PDTS+ = DAS28-ESR ≤ 2.6 and PDTS > 0

DAS-PDTS- = DAS28-ESR ≤ 2.6 and PDTS = 0.

PDTS = power Doppler tenosynovitis, MTX-TT = Methotrexate (treat-to-target), ETN + MTX = Etanercept + Methotrexate
